# The effects of Gamijinhae-tang on elastase/lipopolysaccharide-induced lung inflammation in an animal model of acute lung injury

**DOI:** 10.1186/1472-6882-13-176

**Published:** 2013-07-16

**Authors:** Sung-Hwa Sohn, HaRyeon Jang, Youngeun Kim, Young Pyo Jang, Seung-Hun Cho, Heejae Jung, Sungki Jung, Hyunsu Bae

**Affiliations:** 1 Department of Physiology, College of Oriental Medicine, Kyung Hee University, #1, Hoegi-dong, Dongdaemun-gu, Seoul, 130-701, Republic of Korea; 2 Division of Allergy and Respiratory System, Department of Oriental Internal Medicine, College of Oriental Medicine, Kyung Hee University, #1, Hoegi-dong, Dongdaemun-gu, Seoul, 130-701, Republic of Korea; 3 Division of Pharmacognosy, College of Pharmacy, Kyung Hee University, #1, Hoegi-dong, Dongdaemun-gu, Seoul, 130-701, Republic of Korea; 4 Department of Life and Nanopharmaceutical Science, College of Pharmacy, Kyung Hee University, #1, Hoegi-dong, Dongdaemun-gu, Seoul, 130-701, Republic of Korea; 5 Department of Neuropsychiatry, College of Oriental Medicine, Kyung Hee University, #1, Hoegi-dong, Dongdaemun-gu, Seoul, 130-701, Republic of Korea; 6 Institute of Oriental Medicine, Kyung Hee University, Seoul, 130-701, South Korea; 7 Present Address: National Academy of Agricultural Science, Rural Development Administration, Suwon, 441-707, South Korea

**Keywords:** ALI, COPD, Gamijinhae-tang, Elastase, IL-6, IL-1β, Goblet cell

## Abstract

**Background:**

Gamijinhae-tang (GJHT) has long been used in Korea to treat respiratory diseases. The therapeutic effect of GJHT is likely associated with its anti-inflammatory activity. However, the precise mechanisms underlying its effects are unknown. This study was conducted to evaluate the protective effects of GJHT in a porcine pancreatic elastase (PPE) and lipopolysaccharide(LPS) induced animal model of acute lung injury (ALI).

**Methods:**

In this study, mice were intranasally exposed to PPE and LPS for 4 weeks to induce chronic obstructive pulmonary disease (COPD)-like lung inflammation. Two hours prior to PPE and LPS administration, the treatment group was administered GJHT extracts via an oral injection. The numbers of neutrophils, lymphocytes, macrophages and total cells in the bronchoalveolar lavage (BAL) fluid were counted, and pro-inflammatory cytokines were also measured. For histologic analysis, hematoxylin and eosin (H&E) stains and periodic acid-Schiff (PAS) stains were evaluated.

**Results:**

After inducing ALI by treating mice with PPE and LPS for 4 weeks, the numbers of neutrophils, lymphocytes and total cells were significantly lower in the GJHT group than in the ALI group. In addition, the IL-1β and IL-6 levels were significantly decreased in the GJHT group. The histological results also demonstrated the attenuation effect of GJHT on PPE- and LPS-induced lung inflammation.

**Conclusions:**

The results of this study indicate that GJHT has significantly reduces PPE- and LPS-induced lung inflammation. The remarkable protective effects of GJHT suggest its therapeutic potential in COPD treatment.

## Background

Chronic obstructive pulmonary disease (COPD) is a leading and increasing cause of morbidity and mortality. Worldwide, it is projected to rise to rank fifth in disease burden by 2020, and it now ranks as the third leading cause of death in the USA [1,2]. COPD is characterized by lung parenchyma destruction, mucus hypersecretion, emphysema, an abnormal lung inflammatory response to external stimuli, and progressive airflow limitation [3-5]. The pathophysiology of airways in COPD involves neutrophilic airway inflammation, antiprotease imbalances, and oxidative stress [6]. The major cause of COPD is cigarette smoking [1,7-10]. Tobacco contains high levels of LPS, approximately 1% of which survives combustion and is an active component of cigarette smoke [11,12]. LPS is a major proinflammatory component of gram-negative bacteria. In experimental animals, the exposure of mice to inhaled LPS was shown to cause emphysema-like changes that persisted up to 4 weeks [13]. Recently, Sajjan et al. reported that PPE- and LPS-treated mice exhibited pathological and physiological changes typical of COPD, showing airway hyperresponsiveness (AHR) and increased airway inflammation [14]. In this study, we selected the PPE/LPS-treated animal model to better understand the therapeutic effects of herbal medicines on COPD.

Gamijinhae-tang (GJHT) has been used to treat respiratory diseases such as emphysema, bronchitis, asthma, and COPD in Korea for centuries. GJHT contains 16 species of medicinal plants [15,16]. In Korean traditional medicine, medicinal plants are generally used in mixtures. Medicinal plants have long been used to treat inflammation and other inflammation-related diseases, and the raw materials of these products are often used to develop new drugs [17-20]. However, the preventative effects and underlying mechanisms of GJHT on pulmonary disorders have not yet been evaluated. In this study, we provide evidence that GJHT has potential preventative effects on COPD.

## Methods

### Water extraction of Gamijinhae-tang (GJHT)

Sixteen medical herbs were used to prepare GJHT (Table 1). All herbs were purchased from Omniherb (Omniherb Co., Korea). A total of 258 grams of GJHT was boiled in 1.4 liters of distilled water in an Herb Extractor (Dae-Woong Co., Korea) for 2 hr, yielding a final 580 ml of GJHT extract. The supernatant was harvested under sterile conditions by centrifugation and was lyophilized through evaporation at −80°C. The lyophilized GJHT extract was dissolved in an appropriate volume of sterile PBS prior to its administration to mice.

**Table 1:** Perscription of Gamijinhae-Tang (GJHT)

**Scientific name**	**Amount (%)**	**Chemical marker**
*Rehmannia glutinosa Liboschitz var. purpurea Makino*	14	5-HMF
*Raphanus sativus var. hortensis for. acanthiformis MAKINO*	14	
*Astragalus membranaceus Bunge. var. membranaceus*	9	
*Atractylodes macrocephala Koidz.*	9	
*Poria cocos Wolff*	5	
*Pinellia ternate (Thunb.) Breit.*	5	
*Citrus unshiu Markovich*	5	Hesperidin
*Angelica gigas NAKAI*	5	Decursin
*Liriope platyphylla F. T. Wang* &*T. Tang*	5	
*Platycodon grandiflorum A. De Candolle*	5	
*Anthriscus sylvestris*	5	
*Schisandra chinensis (Turcz.) Baill*	5	
*Morus alba L.*	5	
*Scutellaria baicalensis*	5	Baicalin, Baicalein
*Sinapis semen*	3	
*Glycyrrhiza uralensis FISCH*	3	
Total	100	

### High performance liquid chromatography (HPLC) Analysis of GJHT

5-Hydroxymethylfuraldehyde (5-HMF) was purchased from Sigma Aldrich (Wisconsin, UA), and baicalin, baicalein, hesperidin and decursin were purchased from Wako Chemicals (Osaka, Japan). HPLC grade acetonitrile was purchased from J. T. Baker (NJ, USA). A hundred miligram of GJHT extract was dissolved in 1 ml of methanol and distilled water (1:20) and extracted through C18 SPE cartridge (Waters) to remove poly-saccharides. Residual portions were recovered and dried under nitrogen to yield 16.1 mg of residue. The residue was dissolved in 1 ml of same solvent above. All standards and sample solutions were filtered through a 0.45 mm syringe filter (Millipore) before the injection into the Ultra Performance Liquid Chromatography (UPLC). Chromatographic analysis was performed using an Acquity™ UPLC system (Waters, Milford, MA, USA) comprising a Quaternary Solvent Manager, a Sample Manager – TFN and a PDA detector, and the system was operated by the Waters Empower 3 software. The samples were analyzed with a Acquity UPLC® BEH C18 column (50 × 2.1 mm i.d.; 1.7 μm) whole through the experiment. The PDA data were collected from 200 to 500 nm. The mobile phase consisted of acetonitrile (solvent A) and water (solvent B) in a linear gradient that increased from 1% to 100% of solvent A over 20 minutes. The flow rate was 0.5 ml/min and the injection volume was 2 μl.

### Animals

The protocols presented below are based on animal experiments approved by the Animal Ethics Committee of Kyung Hee University (12–015). Balb/c male mice (6 weeks of age, weighing 20 – 25 g) were purchased from Charles River Korea (Orient Bio, Seungnam, South Korea). All mice were maintained under specific pathogen-free conditions during the experiments, which were performed according to the ethical principles and guidelines established by the Kyung Hee University for the care and use of experimental animals.

### Animal treatment and ALI induction

The mice were randomly divided into five groups (n = 5–7/group): (1) control group: negative control mice were sensitized and challenged with PBS alone; (2) ALI group: mice were exposed by the intranasal route to 1.2 units of porcine pancreatic elastase (PPE; Elastin products, Owensville, MO, USA) on day 1 and 7 μg of LPS (Calbiochem, Germany) on day 4 of the week for 4 consecutive weeks.; (3) dexa group: positive-control mice were administered dexamethasone (1 mg/kg body wt) via an oral injection 2 hr prior to PPE and LPS stimulation; (4) GJHT 100 mg/kg group: mice were administered GJHT (100 mg/kg body wt) via an oral injection 2 hr prior to PPE and LPS stimulation; (5) GJHT 300 mg/kg group: mice were orally administered GJHT (300 mg/kg body wt) 2 hr prior to PPE and LPS stimulation. On day 32, the mice were sacrificed, and various tissues were collected for analyses.

### Analysis of lung inflammatory cells

Phosphate-buffered saline (PBS) was slowly infused into the lungs and then withdrawn via a cannula that had been inserted into the trachea. The numbers of total and differential cells in the bronchoalveolar lavage (BAL) fluid were then determined using a hemacytometer. In addition, differential cell counts were conducted on slides that were prepared by cytocentrifugation and Diff-Quick staining. Approximately 500 cells were counted per slide. The BAL fluids were then centrifuged, and the supernatants were stored at −80°C until needed.

### Measurements of IL-1β and IL-6 in BAL fluid

The levels of IL-1β and IL-6 in the BAL fluid were determined using a commercial enzyme immunoassay kit (BD Pharmingen, USA) according to the manufacturer’s protocols. A 96-well microtiter plate (Costar, NY, USA) was incubated overnight at 4°C with anti-mouse IL-1β and IL-6 monoclonal antibodies in coating buffer and washed with PBS containing 0.05% Tween 20 (Sigma, MO, USA). The IL-1β and IL-6-coated plates were blocked with 5% FBS in PBS for 1 hr at 4°C and with 1% BSA in PBS for 1 hour at room temperature, respectively. BAL fluids (100 μl) were then added and incubated for 2 hr at room temperature. Secondary peroxidase-labeled biotinylated anti-mouse IL-1β and IL-6 monoclonal antibodies were then added and incubated for 1 hour. Finally, the plates were treated with the TMB substrate solution (KPL, CA, USA) for 30 min, and the reaction was stopped with the addition of the TMB stop solution (50 μl per well). The optical density was measured at 450 nm in a microplate reader (SoftMax PRO, version 3.1 software, CA, USA). The detection limit for IL-1β and IL-6 was 15.6 pg/ml.

### Preparation of lung tissues and histology and immunohistochemistry

Lung tissues were fixed in a 4% paraformaldehyde solution and then embedded in paraffin. For histological examination, 4-μm sections of lung tissue were stained sequentially with hematoxylin and eosin (H&E) or periodic acid-Schiff (PAS). To immunohistochemically evaluate neutrophil elastase (NE) and proliferating cell nuclear antigen (PCNA), 4-um sections of lung tissue were treated with 0.3% H_2_O_2_-methanol for 30 min to block endogenous peroxidases, after which they were incubated at 4°C overnight with anti-NE goat polyclonal antibody (1:50 dilution; Santa Cruz Biotechnology, CA, USA) or anti-PCNA rabbit polyclonal antibody (1:50 dilution; Santa Cruz Biotechnology, CA, USA). The slides were then incubated with avidin-biotin peroxidase complex (ABC kit, Vector Laboratories, CA, USA), after which the color was developed with 3,3′-diaminobenzidine tetrachloride (DAB, Vector Laboratories, CA, USA). Following immunohistochemical staining, the slides were counterstained with Herris’s hematoxylin for 2 min and then mounted with Canada balsam (Showa Chemical Co. Ltd., Japan). Sections with maximum cross-section of parenchyma were selected for morphometry using digitized image analysis. Images were digitized and evaluated with Image Pro-Plus 5.1 software (Media Cybernetics, Inc. Silver Spring, MD, USA). Mean alveolar airspace was determined from the sum of the lumen divided by the number of identified alveoli.

### RNA preparation

The RNA was then isolated from the lung tissue using an Rneasy® Mini Kit (Qiagen, CA, USA) according to the manufacturer’s instructions, and the RNA was quantified using NanoDrop (ND-1000; NanoDrop Technologies, Inc., Wilmington, DE, USA).

### Real-time RT-PCR analysis

PAS staining verification was performed via Real-time RT-PCR analysis of Muc5AC and Muc5B genes using LightCycler® 480 SYBR Green I Master mix and Light Cycler 480 real-time PCR machine (Roche Applied Science, Indianapolis, IN, USA). Expression levels of transcripts were evaluated using the comparative CT method (2-deltaCT). Transcript levels of GAPDH were used for sample normalization. Results are log2-transformed fold changes of normalized 2-deltaCT. Data were obtained from three independent experiments and are represented as average ± standard error. The sequences of the mouse primers were as follows: Muc5AC (FW 5′-cgctaacctgccaaaagaag-3′; RW 5′-gctgaactggggacaacatt-3′), Muc5B (FW 5′-ccgtcctctttcccaacata-3′; RW 5′-ttggttgtcactctgcttgc-3′), and GAPDH (FW 5′-ttcaccaccatggagaaggc-3′; RW 5′-ggcatggactgtggtcatga-3′).

### Statistical analysis

Statistical analysis of the data was conducted using Prism 5 software (GraphPad Software Inc., San Diego, CA, USA). All values are presented as the mean ± S.E.M. Differences between the means of the control and treatment samples were determined by an one-way ANOVA followed by Newman-Keuls *post*-*hoc* test. Results with a p < 0.05 were considered statistically significant. The power calculation was conducted from one-way ANOVA power analysis based on effect size (SPSS, IBM, Armonk, NY, USA). The power (1-β) was 0.96 from one-way ANOVA power analysis (α error = 0.05,effect size f =0.97). Therefore total sample size (n = 26) was enough to allow for statistically significant finding.

## Results

### The HPLC profile of GJHT

**Figure 1:**
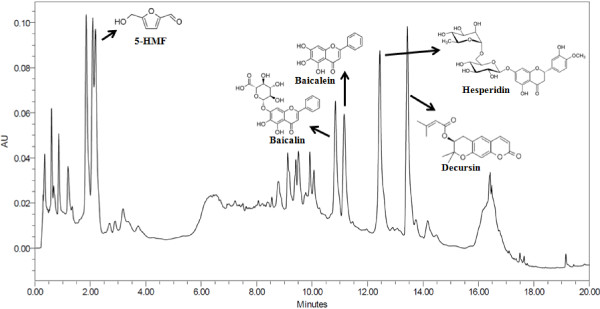
**The UPLC profile of Gamijinhae-tang (GJHT) extract monitored at 280 nm.** Identified peaks and corresponding standard compounds were indicated on the UPLC chromatogram.

The identified compounds of GJHT using UPLC were listed Table 1. Five representative chemicals were clearly identified in UPLC chromatograph (Figure 1). Identified peaks and corresponding standard compounds were indicated on the UPLC chromatogram (Figure 1).

### The effect of GJHT on pulmonary inflammation

**Figure 2:**
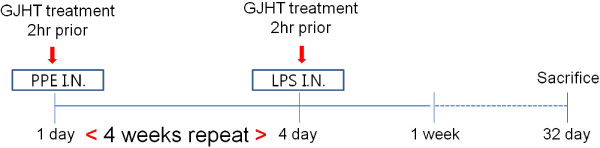
**Schematic diagram of the experimental protocol.** Animals were exposed by intranasal route to 1.2 U/kg of porcine pancreatic elastase (PPE) on day 1 and 7 ug/kg of lipopolysaccaride (LPS) on day 4 of the week for 4 consecutive weeks. The mice were sacrificed on 7 days at after last LPS stimulation.

**Figure 3:**
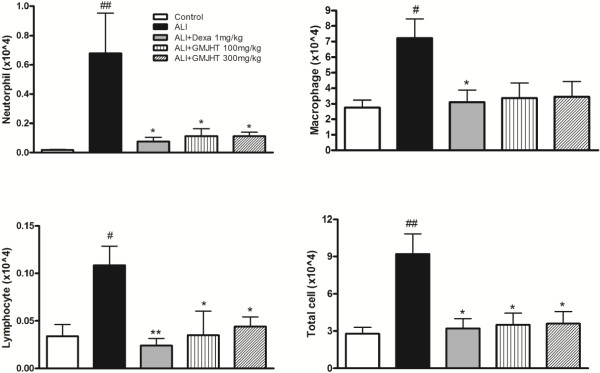
**Effect of Gamijinhae-tang (GJHT) extract on immune cell profiles in BAL fluid.** The number of neutrophils (p = 0.042, F = 3.00, and R^2^ = 0.36), macrophages (p = 0.0145, F = 4.00, and R^2^ = 0.43), lymphocytes (p = 0.0049 F = 5.00, and R^2^ = 0.49), and total cells (p = 0.0016, F = 6.68, and R^2^ = 0.58) were determined in BAL fluid. Control: saline treated, ALI: PPE (porcine pancreatic elastase) + LPS (lipopolysaccaride) treated, ALI + Dexa: ALI + dexamethasone (Dexa), ALI + GJHT: ALI + GJHT (Gamijinhae-tang). Data are expressed as the mean number of cells ± S.E.M. (# p < 0.05, ## p < 0.01 versus control and *p < 0.05, **p < 0.01 versus ALI; n = 5-7).

To determine whether GJHT affects immune cells, mice were subjected to a long-term exposure to PPE and LPS (four weeks, Figure 2). At one week after the final LPS treatment, a significant increase in the total number of cells was observed in the ALI group when compared to the dexamethasone-treated (1 mg/kg body wt) and GJHT-treated (100 or 300 mg/kg body wt) groups (Figure 3). In addition, the influx of macrophages, neutrophils, and lymphocytes was remarkably higher in the ALI group than in the dexamethasone-treated (1 mg/kg body wt) and GJHT-treated (100 or 300 mg/kg body wt) groups (Figure 3).

### The effects of GJHT on pro-inflammatory cytokine production in BAL fluid

**Figure 4:**
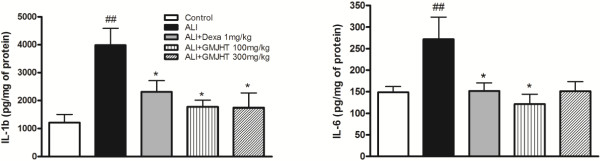
**Effect of Gamijinhae-tang (GJHT) extract on cytokine in BAL fluid.** The levels of IL-1b and IL-6 in BAL fluid were determined by ELISA. Control: saline treated, ALI: PPE (porcine pancreatic elastase) + LPS (lipopolysaccaride) treated, ALI + Dexa: ALI + dexamethasone (Dexa), ALI + GJHT: ALI + GJHT (Gamijinhae-tang). Data are expressed as the mean ± S.E.M. (## p < 0.01 versus control and *p < 0.05, **p < 0.01 versus ALI; n = 5-7).

To evaluate the effects of GJHT on BAL fluid, the secretion of pro-inflammatory cytokines was measured. IL-1β and IL-6 are known to be pro-inflammatory cytokines that contribute to LPS-induced lung inflammation. Treatment with GJHT significantly reduced the levels of IL-1β and IL-6 when compared to the ALI group (IL-1β; p = 0.0029, F = 5.67, R^2^ = 0.52, and IL-6; p = 0.032, F = 3.23, R^2^ = 0.38, Figure 4).

### The effect of GJHT on histological changes in lung tissue

**Figure 5:**
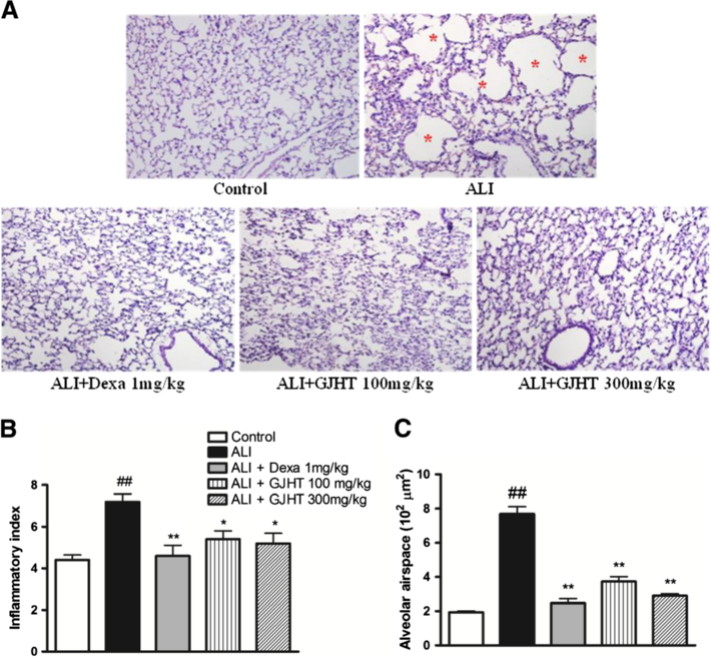
**The effect of Gamijinhae-tang (GJHT) extract on lung tissue damage. A)** Mouse lung sections were stained with hematoxylin and eosin (magnification × 200), **B)** inflammatory index (p = 0.001, F = 7.14, and R^2^ = 0.59), and **C)** Alveolar airspace. Control: saline treated, ALI: PPE (porcine pancreatic elastase) + LPS (lipopolysaccaride) treated, ALI + Dexa: ALI + dexamethasone (Dexa), ALI + GJHT: ALI + GJHT(Gamijinhae-tang). Data are expressed as the mean ± S.E.M. (## p < 0.01 versus control and *p < 0.05, **p < 0.01 versus ALI; n = 5-7).

We also evaluated the effects of GJHT on PPE- and LPS-induced lung damage. We stained lung sections with hematoxylin and eosin (H&E). We found that lung architecture of ALI group was distinct from controls with respect to alveolar airspace. The ALI group showed alveolar destruction, which resulted in enlarged air spaces, indicating an emphysematous change. By contrast, the dexamethasone-treated (1 mg/kg body wt) and GJHT-treated (100 or 300 mg/kg body wt) groups showed less tissue damage (p < 0.0001, F = 69.73, and R^2^ = 0.94, Figure 5).

### The effect of GJHT on goblet cell hyperplasia

**Figure 6:**
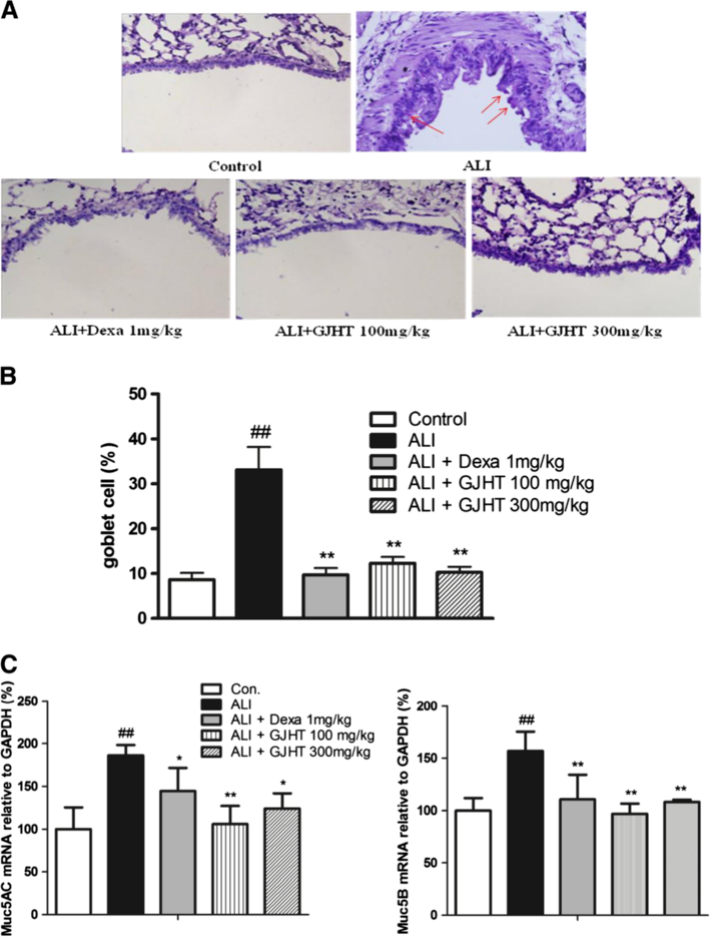
**The effect of Gamijinhae-tang (GJHT) extract on goblet cell and musins. A)** Mouse lung sections were stained with PAS (magnification × 400), **B)** goblet cell (%), and **C)** Muc5AC and Muc5B mRNA expression. Muc5AC and Muc5B mRNA expression were analyzed by quantitative real-time PCR. Expression levels of Muc5AC and Muc5B mRNA were normalized to GAPDH and expressed as the relative quantity to controls. Control: saline treated, ALI: PPE (porcine pancreatic elastase) + LPS (lipopolysaccaride) treated, ALI + Dexa: ALI + dexamethasone (Dexa), ALI + GJHT: ALI + GJHT (Gamijinhae-tang). Data are expressed as the mean ± S.E.M. (## p < 0.01 versus control and *p < 0.05, **p < 0.01 versus ALI; n = 5-7). The statistic power for Muc5AC; p = 0.0039, F = 7.89, and R^2^ = 0.76, and for Muc5B; p = 0.0042, F = 7.70, and R^2^ = 0.75.

To evaluate the effects of GJHT on goblet cell hyperplasia in lung tissue, the number of PAS-positive cells was evaluated. The ALI group showed more PAS-positive goblet cells in the large airways. By contrast, dexamethasone-treated (1 mg/kg body wt) or GJHT-treated (100 or 300 mg/kg body wt) groups showed significantly fewer PAS-positive cells than ALI group (p < 0.0001, F = 15.94, and R^2^ = 0.76, Figure 5). PAS staining verification was performed via Muc5AC and Muc5B mRNA expression. The mucins Muc5AC and Muc5B are found at increased levels in ALI group. However, dexamethasone-treated (1 mg/kg body wt) or GJHT-treated (100 or 300 mg/kg body wt) groups are decreased expression than ALI group (Figure 6). These data suggest that treatment with GJHT had a powerful preventative effect on the induced chronic inflammatory lung disease.

## Discussion

Exposures to environmental allergens are the main triggers of asthma, while cigarette smoking is the predominant cause of COPD [21]. Both asthma and COPD are characterized by chronic airway inflammation. The key inflammatory cells involved in COPD (CD8^+^ T cells, macrophages and neutrophils) are different from those involved in asthma (eosinophils and CD4^+^ Th2 cells), suggesting that different treatments may be required [9,22]. However, the treatments for both of these diseases may often overlap. The current standard medications for treating inflammatory lung diseases has evolved to cocktail therapies, which are combinations of several medicines possessing different therapeutic targets and include inhaled glucocorticosteroids, β2-adrenoceptor agonists, leukotriene receptor antagonists, methylxanthines, theophylline, and others [23-26]. However, these therapies produce potential side effects, such as growth retardation, the induction of insulin resistance, the loss of bone mass, immune system suppression, cardiac comorbidity, nausea, emesis, gastrointestinal disturbances, and arrhythmias, but they do not consistently ameliorate airway inflammation in some COPD patients [27-33]. Therefore, there is a need for the development of safe and efficacious treatments [34,35]. In the present study, we show that exposure to PPE and LPS induces structural and functional changes that are typical of COPD, including airway remodeling, diffuse lung inflammation, goblet cell hyperplasia, alveolar enlargement, and increased numbers of neutrophils, lymphocytes, and macrophages in the airways and alveoli. In addition, in the airways the mucins Muc5AC and Muc5B are found at increased levels in both asthmatic and COPD subjects [36,37]. Mucin hypersecretion is associated with abnormal epithelial cell growth and differentiation, both inflammatory mediators and growth factors may be involved in the stimulation of mucin production from goblet cells [38]. However, GJHT treatment ameliorated the lung structural and functional changes in the PPE and LPS exposure model. These results may suggest that GJHT is a useful therapeutic agent that prevents the structural and functional changes associated with COPD.

The components of GJHT have been shown to have various biological effects. *Rehmannia glutinosa*, a major component of GJHT, possesses thrombolytic, hyperglycemic, and anti-inflammatory activities and improves renal functions in diabetic nephropathy [39-43]. In addition, this herb has been reported to suppress the production of TNF-α and IL-1 in mouse astrocytes [44]. *Atractylodes macrocephala*, another component of GJHT, possesses antioxidant, hepatoprotective, anti-inflammatory, anti-allergic, antithrombotic, antiviral and anticarcinogenic activities [45-47]. *Angelica sinensis* is well known to have strong immune regulatory effects, such as antiviral activities and improving immune function by increasing CD4^+^ cells and the CD4^+^/CD8^+^ ratio [48]. *Liriope platyphylla* has been used to treat asthma and bronchial and lung inflammation [49]. In addition, this compound possesses various therapeutic effects for conditions such as obesity, diabetes, inflammation and neurodegenerative disease [50-53]. *Scutellaria baicalensis* has been used to treat inflammation, cancer, bacterial and viral infections of the respiratory and gastrointestinal tracts [54-56]. Some of the components of GJHT are reported to possess anti-cancer effects for various types of malignancy via distinct molecular mechanisms and include *Raphanus sativus*[57,58], *Astragalus membranaceus*[59-61], *Poria cocos*[62], *Citrus unshiu*[63], *Platycodon grandiflorum*[64] and *Anthriscus sylvestris*[65]. The therapeutic potency of GJHT should be attributed to its combined and synergistic effects on multiple targets as a result of the diverse components of GJHT. In addition, according to the formula suggested by US Food and Drug Administration [66], human equivalent dose (HED) calculated from mice dosage (100 mg/kg and 300 mg/kg) used in this study was 480 mg/60 kg and 1440 mg/60 kg in human, respectively. We believe that these amounts of GJHT would be applicable dosages to human.

The morphological and inflammatory changes were accompanied by increases in lung IL-1β and IL-6, as observed in humans with COPD [67,68]. A recent genetic analysis study demonstrated that *IL6, IL1RN, IL1B*, and *IFNG* genes were risk factors for the accelerated decline of lung function or baseline lung function in COPD patients [69]. Importantly, their study demonstrated a significant association between *IL6* and an *IL6*-smoking interaction in cardiovascular disease [69]. In addition, high levels of serum or sputum IL-6 have been associated with impaired lung function, pulmonary infections, exacerbations, and skeletal muscle weakness in COPD patients [70-74]. Experimental studies have shown that IL-6 overexpression in the murine lung results in emphysema-like airspace enlargement and airway inflammation [75]. In the present study, GJHT treatment significantly reduced the amounts of IL-1β and IL-6 in the airway, suggesting that the anti-inflammatory effect of GJHT can be attributed to the suppression of proinflammatory cytokine production in the lung. Even though the power calculation result demonstrated that the animal number used in this study was enough to tell the statistical importance, the sample size of this study was relatively small (n = 5,6 each group) that might mislead the data interpretation. To overcome such limitations, additional researches investigating the mechanism of Gamijinhae-tang on lung inflammation are necessary.

## Conclusions

Taken together, the results of this study suggest that GJHT significantly reduces PPE- and LPS-induced lung inflammation. The anti-inflammatory effects of GJHT indicate that it has therapeutic potential for chronic obstructive pulmonary disease.

## Competing interests

The authors declare that they have no competing interests.

## Authors’ contribution

SS, HJ and YK have made contribution to acquisition and analyzing data. YPJ, SC and HJ have made been involved in interpretation of data. SS, SJ and HB have been involved in designing the study and drafting the manuscript. All authors read and gave final approval for the version submitted for publication.

## Pre-publication history

The pre-publication history for this paper can be accessed here:

http://www.biomedcentral.com/1472-6882/13/176/prepub
